# Impacts of Covid-19 interventions on air quality: evidence from Brazilian metropolitan regions

**DOI:** 10.1007/s13762-022-04189-6

**Published:** 2022-05-03

**Authors:** F. C. Silva, D. H. Silva, K. M. Zamprogna, S. S. Souza, D. Sell, J. Sabatini-Marques, T. Yigitcanlar

**Affiliations:** 1 College of Administration and Economic Science, State University of Santa Catarina, Av. Madre Benvenuta, 2007, Itacorubi, Florianópolis, SC 88035-901 Brazil; 2 Department of Environmental Criminal Forensics, Federal University of Santa Catarina, Campus Universitario, Trindade, Florianópolis, SC 88040-900 Brazil; 3 Department of Nursing, School of Health Sciences, Federal University of Santa Catarina, Campus Universitario, Trindade, Florianópolis, SC 88040-900 Brazil; 4 Department of Engineering and Knowledge Management, School of Technology, Federal University of Santa Catarina, Campus Universitario, Trindade, Florianópolis, SC 88040-900 Brazil; 5 School of Architecture and Built Environment, Queensland University of Technology, 2 George Street, Brisbane, QLD 4000 Australia

**Keywords:** Air quality, Brazil, Climate change, Covid-19, Nitrogen dioxide, Urban policy

## Abstract

The Covid-19 pandemic has negatively disrupted the way our economy and society functions. Nonetheless, there have also been some positive externalities of the pandemic on the environment. This paper aims to evaluate the concentration of nitrogen dioxide in Brazilian metropolitan regions after the policies adopted to confront Covid-19. In terms of methodological approach, the study employs cross-sectional quantitative analyses to compare the period of 36 days, i.e., 12 March to 16 April—before (in 2019) and after (in 2020) the pandemic declaration. The data were obtained from the Sentinel 5-P low-Earth polar satellite concerning Brazilian metropolitan regions (*n* = 24). Thorough spatial and statistical analyses were undertaken to identify the pre- and during pandemic nitrogen dioxide concentrations. Complementarily, Spearman’s correlation test was performed with variables that impact air quality. The study results a fall in nitrogen dioxide concentration levels in 21 of the 24 metropolitan regions which was observed. The Spearman’s correlation coefficient between the nitrogen dioxide variation and the vehicle density was 0.485, at a significance level of 0.05. With these findings in mind, the paper advocates that while the pandemic has a significant negative consequence on the health of population globally, a series of measures that result in a new social organization directly interfere in the reduction of air pollution that contributes to the quality of the air we breathe.

## Introduction

Nitrogen dioxide (NO_2_), a gas resulting mainly from activities of fossil fuels burning, such as vehicle transport and industrial production, is polluting in high atmospheric concentration, that is, it leads to environmental pollution (Mahbub et al. 2011). However, a scenario of actions and attitudes aimed at containing Covid-19 pandemic led to new behaviors and political attitudes, which directly interfered with the atmospheric concentration of NO_2_ (Araujo-Filho et al. 2020).

The current Covid-19 pandemic is related to an acute respiratory disease caused by a new coronavirus (SARS-CoV-2), which is highly contagious, and its evolution is still little known (Brasil 2020). Due to the size and speed at which the infection spreads worldwide, its recovery or control required to work on a global and national scale that, due to local and regional diversities, led countries to plan and take action in different ways, as observed in the current sanitary situation of Covid-19 pandemic many countries (Yigitcanlar et al. 2022)—including Brazil.

Nonetheless, the recent changes in the diagnostic criteria of the disease have led to an increase in the rate of new cases and, every day, increasing numbers and challenges have been the subject of intense debate in the scientific community (Araujo-Filho et al. 2020; Brasil 2020). Despite the recent availability of vaccines, still transmission of SARS-CoV-2 is possible. Hence, the World Health Organization (WHO) recommends social distancing, respiratory etiquette, and hand hygiene as the most efficient measures to combat the pandemic, also called non-pharmacological. In this sense, on January 22, 2020, the Ministry of Health introduced a series of measures for the Brazilian population, such as guidelines on hand hygiene and social distancing (Ogen 2020). Social distancing actions were widely publicized by local governments and have social network as an ally, which could expand the dissemination of necessary measures; it is also a tool to guide government on its decision making (Yigitcanlar et al. 2020). Therefore, ministerial recommendations allied to state governments attitudes led to a decrease in transit, industrial actions, among other activities that, due to social isolation, decreased the concentration of pollutants in the atmosphere (Kroll et al. 2020).

In a study carried out in 10 countries (Australia, Brazil, China, Ghana, India, Iran, Italy, Norway, South Africa, and the USA), people declared they have noticed an improvement in the quality of the breathed air after the adoption of social isolation in their regions (Barbieri et al. 2020). Nevertheless, it is not just a matter of noticing the reduction, but of relevant diagnosed improvements in global air quality, with reduction in NO_2_ concentrations, carbon monoxide (CO), thin particulate material (PM_2,5_), and increased ozone (O_3_) concentration in different regions of the globe (Venter et al. 2020; Liu et al. 2021).

In China, the country where SARS-CoV-2 had its origin and where the isolation measures were started in January 2020, the reduction of atmospheric pollutants, such as NO_2_ (Venter et al. 2020; Wang et al. 2020; Chen et al. 2020; Chen et al. 2021) and PM_2,5_ (Venter et al. 2020; Chen et al. 2020; Giani et al. 2020; Shi et al. 2021), has been verified after the adoption of measures to cope Covid-19. In Southeast Asia, air quality during the pandemic period was also object of study, with improvement in cities like India (Mahato et al. 2020; Thomas et al. 2020; Tyagi et al. 2021). In Pakistan, on the other hand there were no significant changes in PM_2,5_ (Mehmood et al. 2021).

The same phenomenon of reduction in the concentration of atmospheric NO_2_during measures to confront Covid-19 was observed in Europe, in German cities (Ogen 2020; Liu et al. 2021; Burns et al. 2021), Italy (Ogen 2020; Liu et al. 2021; Cameletti 2020), France (Ogen 2020; Liu et al. 2021), Spain (3, Liu et al. 2021), and the UK (Liu et al. 2021; Jephcote et al. 2021; Wyche et al. 2021). The reduction of concentrations of PM_2,5_ in the same period was also found in cities of France (Liu et al. 2021; Giani et al. 2020; Shi et al. 2021; Connerton et al. 2020), the UK (Liu et al. 2021; Giani et al. 2020; Shi et al. 2021; Jephcote et al. 2021), Germany and Spain (Liu et al. 2021; Giani et al. 2020; Shi et al. 2021).

In the American continent, large cities in the USA also registered decreased level of PM_2,5_, CO, and atmospheric NO_2_ (Shi et al. 2021; Connerton et al. 2020). In Brazil, the focus of this study, São Paulo (Connerton et al. 2020; Debone et al. 2020; Nakada and Urban 2020) and Rio de Janeiro (25), noticed changes in air quality. In the capital of the state of São Paulo, NO_2_ concentration reduced up to 60% if the pandemic period was compared to previous ones (Connerton et al. 2020; Debone et al. 2020; Nakada and Urban 2020).

Against this backdrop, this study aims to evaluate the concentration of NO_2_ in Brazilian metropolitan regions after the social distancing and isolation policy adopted to confront the Covid-19 pandemic. NO_2_ is a chemical that, depending on its concentration, increases air pollution levels, directly influencing on the health of the population and increasing respiratory comorbidities (3,5). In a complementary way, the study focused on evaluating the association of other variables that can directly impact the concentration of atmospheric NO_2_, such as the density of vehicles, especially of combustion engines, in the Brazilian metropolitan regions (Kroll et al. 2020; Derísio 2017; He et al. 2020). The novelty of this paper is being the very first study, in the context of Brazil, investigating atmospheric NO_2_, concentrations by using satellite data and thorough spatial and statistical analyses.

This study was carried out in Brazil in April 2021, with data referring to the period of March 12, 2020, one day after the WHO pandemic state decree, to April 16, 2020, when actions such as social isolation reduced traffic of people and the consequent decrease in industrial production were intensified in Brazil, to avoid Covid-19 (Brasil 2020).

## Materials and methods

This is a quantitative, descriptive, cross-sectional, and documentary study. Descriptive and cross-sectional research combined means that the research object can fully studied at a given historical moment (Sampieri and Collado 2013).

The satellite images used to obtain the tropospheric concentration of NO_2_ were obtained by the polar satellite of low-Earth orbit Sentinel 5-P, of the European Space Agency (ESA–European Space Agency), whose purpose is to obtain information and services of air quality, ozone layer, and climate (TROPOMI 2018). The satellite is equipped with the tropospheric monitoring instrument (TROPOMI—Tropospheric Monitoring Instrument), an image spectrometer that covers ranges of ultraviolet (UV—ultraviolet), visible (VIS—visible), near infrared (NIR), and short-wave infrared (SWIR), using passive remote sensing techniques to measure, at the top of the atmosphere, the reflected and irradiated solar radiation of the Earth (Veefkind et al. 2012).

Under this configuration, TROPOMI is capable of measuring concentrations of ozone (O_3_), nitrogen dioxide (NO_2_), sulfur dioxide (SO_2_), carbon monoxide (CO), methane (CH_4_), formaldehyde (HCHO), and aerosols (Veefkind et al. 2012). Information of NO_2_ tropospheric column is available since April 30, 2018, with a current spatial resolution of 3.5 × 5.5 km (de Vries 2016).

The images were processed in the API (Application Programming Interface) of Google Earth Engine, from where it is possible to obtain the mean and standard deviation of NO_2_ concentrations after overlapping the images, after defining the polygons of interest, which in this study are based on the territorial delimitation of metropolitan regions (Gorelick et al. 2017).

The images used were those recent and high-resolution images of NO_2_ concentrations in the troposphere, also called Near Real-time High-resolution Imagery (NRTI), distributed by ESA and post-processed by the Google Earth Engine API. It is worth noting that daily climatic events, such as the presence of clouds, reduce the quality of images and can influence reading at the time of the satellite overpass. For this purpose, only satellite images with pixels of quality above 75% were used, that is, with the attribute “qa_value” greater than 0.75, in order to avoid erroneous interpretations affected by data quality (Veefkind et al. 2012; Gorelick et al. 2017).

With the set of orbital images selected, the ee.reduce algorithm was used to average all overlapping pixels over the days of the analyzed period (Gorelick et al. 2017) and then reducing data collection to a single image for each of the periods (pre- and post-isolation). This code is shown in Appendix A. After generated, the two images were cut based on the extent of the metropolitan regions and submitted to the calculation of the mean and standard deviation.

Additionally, for maps making, we used on the images the resampling for the resolution of 1000 m and the technique of linear convolution, type Kernel low-pass filter of 3 × 3 pixels, which allows smoothing details and reducing noise of satellite images (Gorelick et al. 2017).

The maps of this 36-day average time were compared to maps from the same period in 2019 to assess the change in NO_2_ emission. For the definition of the study population and in order to delimit its inclusion characteristics, we considered geographic spaces and their territorial dimensions corresponding to the largest Brazilian metropolitan regions, according to the Institute of Applied Economic Research (IPEA 2020), with 24 regions participating in the study.

Once the NO_2_ concentration variations were obtained, these data were compared with the population and vehicle densities of the metropolitan regions, in addition to the variation in the ozone (O_3_) concentration. The population data of the municipalities that make up the metropolitan regions were obtained on the website of the Brazilian Institute of Geography and Statistics (IBGE 2020), taking into account the population estimate of 2020, in order to obtain the ratio of inhabitants per kilometer square of each region.

The density of vehicles in the metropolitan regions was calculated by the relationship between the fleet of licensed vehicles in the municipalities that make up the metropolitan region in April 2020 and the area of each metropolitan region. The fleet of licensed vehicle was obtained from the website of the National Transportation Department (DENATRAN 2020) of the Ministry of Infrastructure and the metropolitan region area was obtained from Institute of Applied Economic Research (IPEA) (IPEA 2020), in order to obtain the ratio of number of vehicles per kilometer square.

The variation in the concentration of O_3_ was obtained by the same method used to define the concentration of NO_2_, previously described. To examine the monotonic association between the variables, the SPSS software was used, where Spearman’s correlation test was performed. The test was chosen in order to assess the strength of the correlation between the variables, and the association results generated were plotted in a table and interpreted at a significance level of 0.05 (Schober et al. 2018).

## Results and discussion

**Fig. 1:**
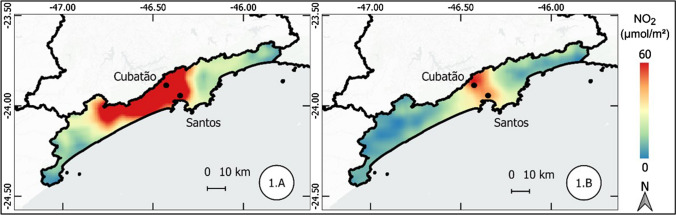
Thematic maps of the mean concentration of NO2 from March 12 to April 16, 2019 and 2020, in Baixada Santista metropolitan region

**Fig. 2:**
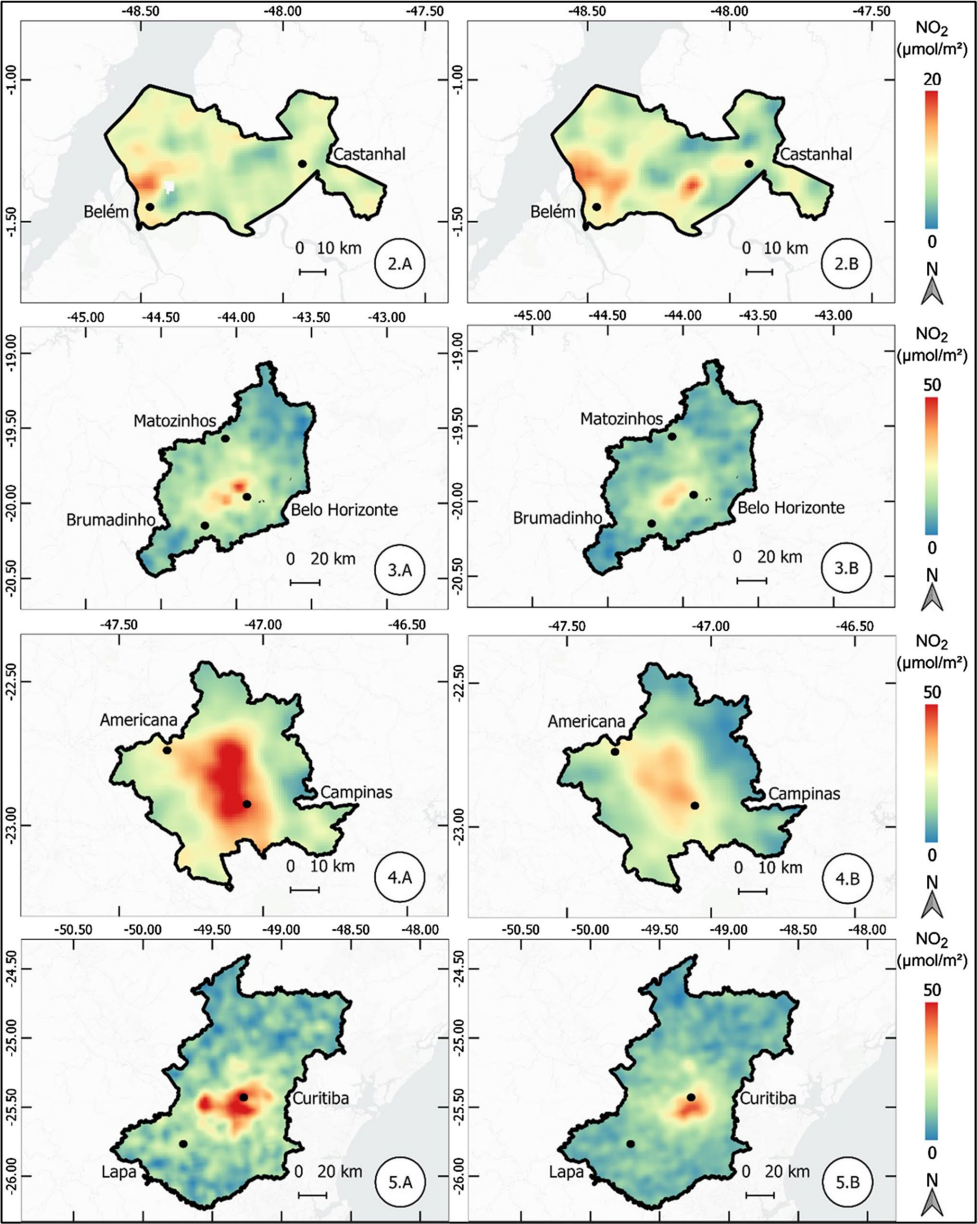
Thematic maps of the mean concentration of NO_2_ from March 12 to April 16, 2019 and 2020, in the metropolitan regions of Belém, Belo Horizonte, Campinas, and Curitiba

**Fig. 3:**
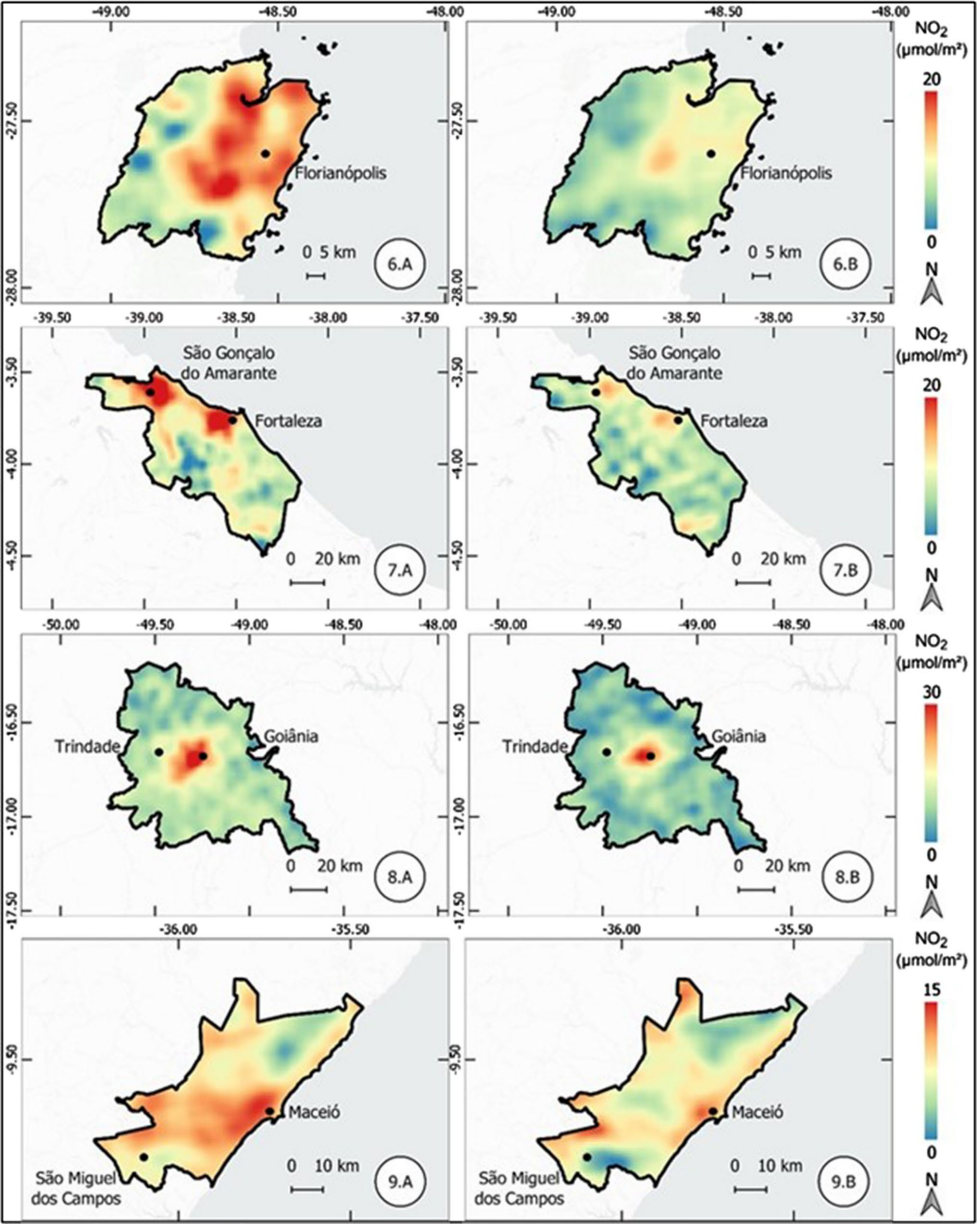
Thematic maps of the mean concentration of NO_2_ from March 12 to April 16, 2019 and 2020, in the metropolitan region of Florianópolis, Fortaleza, Goiânia, and Maceió

**Fig. 4:**
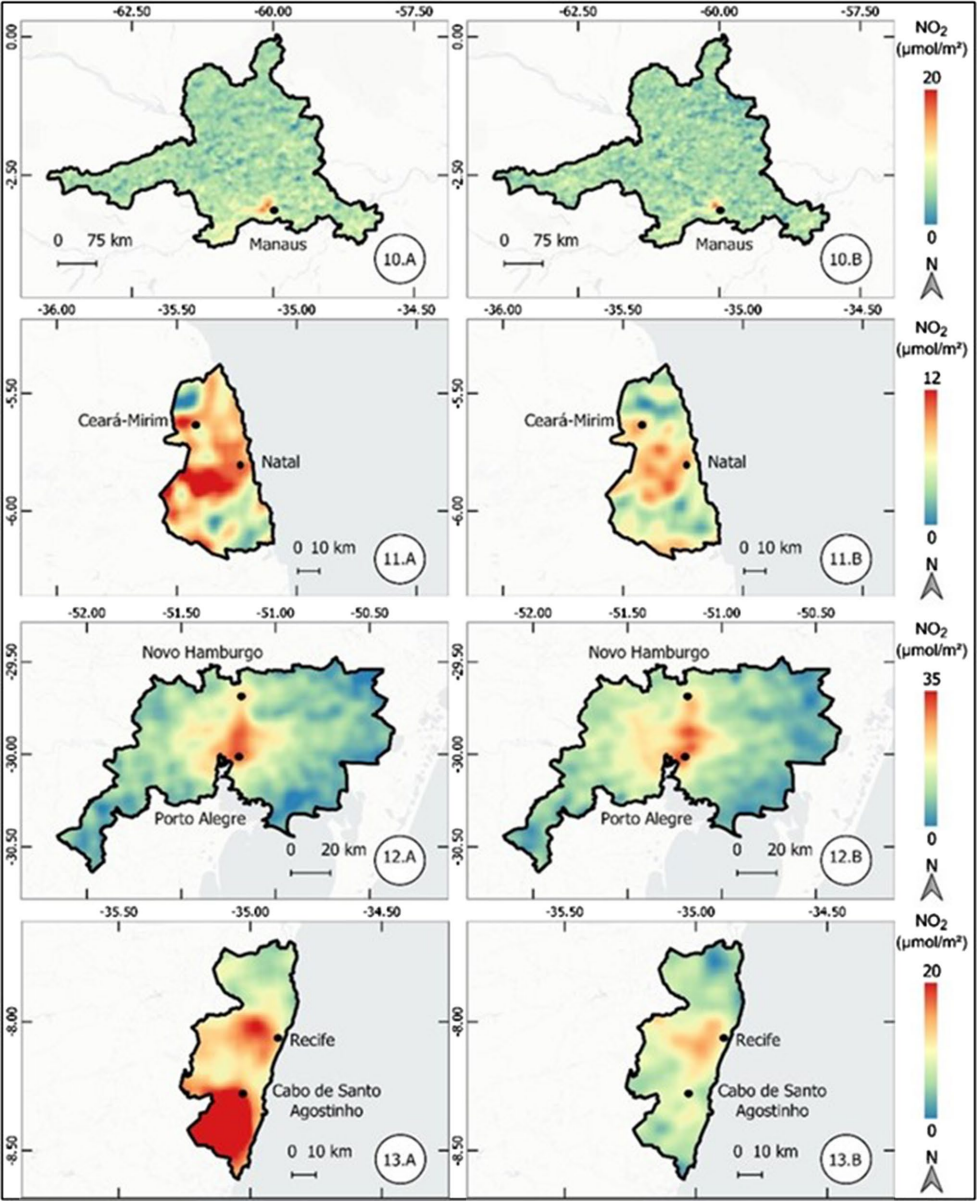
Thematic maps of the mean concentration of NO_2_ from March 12 to April 16, 2019 and 2020, in the metropolitan regions of Manaus, Natal, Porto Alegre, Recife

**Fig. 5:**
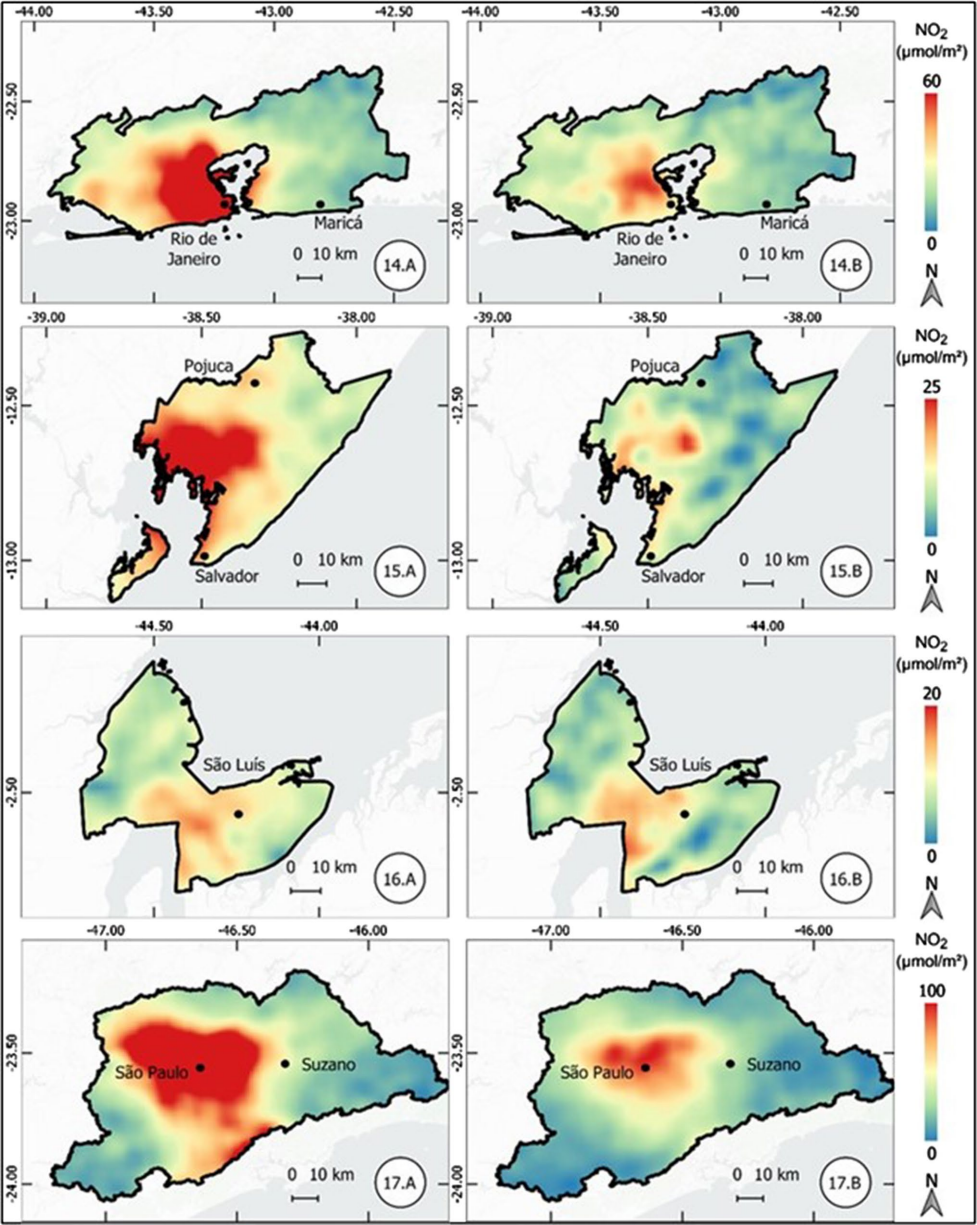
Thematic maps of the mean concentration of NO_2_ from March 12 to April 16, 2019 and 2020, in the metropolitan regions of Rio de Janeiro, Salvador, São Luís, and São Paulo

**Fig. 6:**
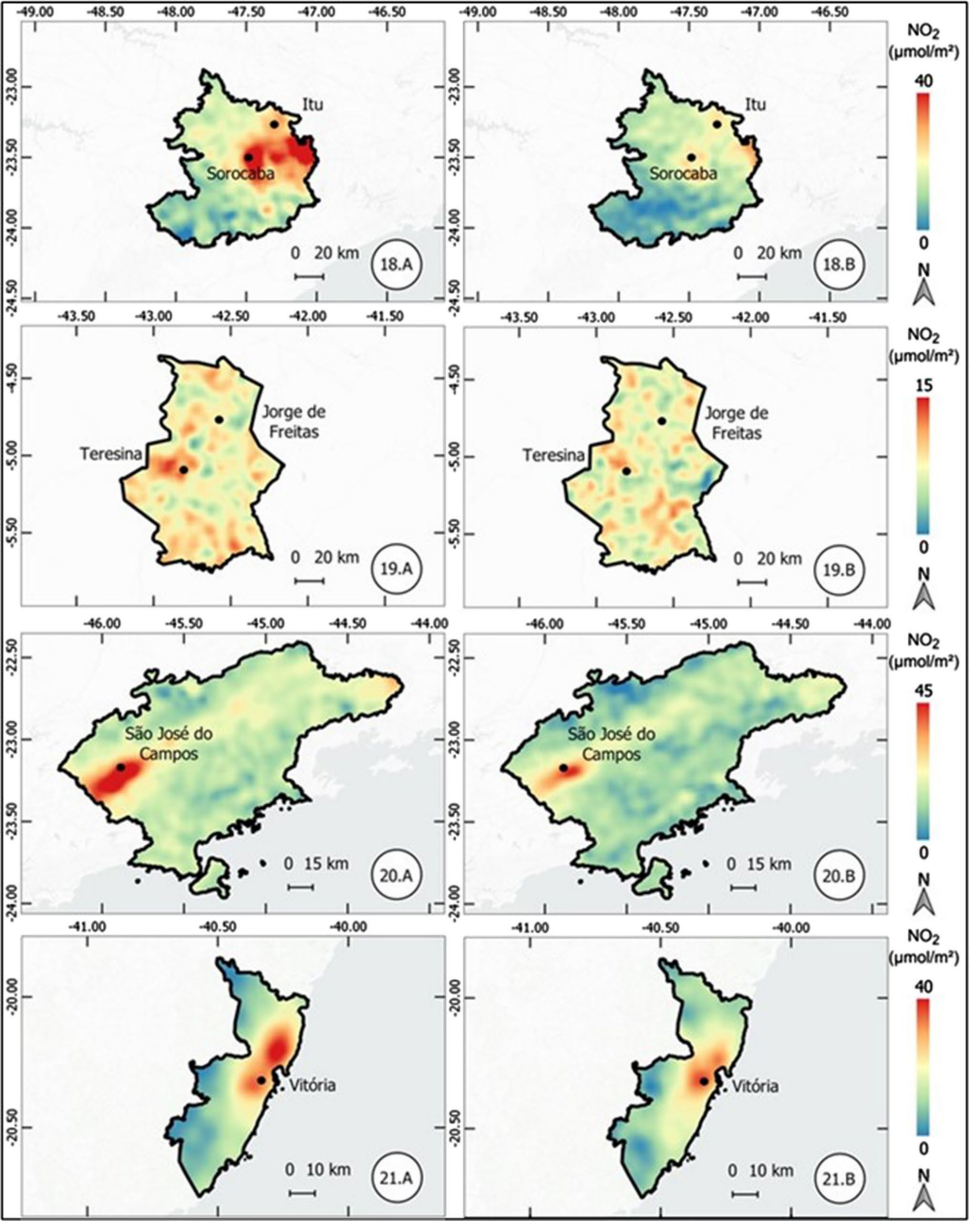
Thematic maps of the mean concentration of NO_2_ from March 12 to April 16, 2019 and 2020, in the metropolitan regions of Sorocaba, Teresina, São José dos Campos (Vale do Paraíba), and Vitória

**Fig. 7:**
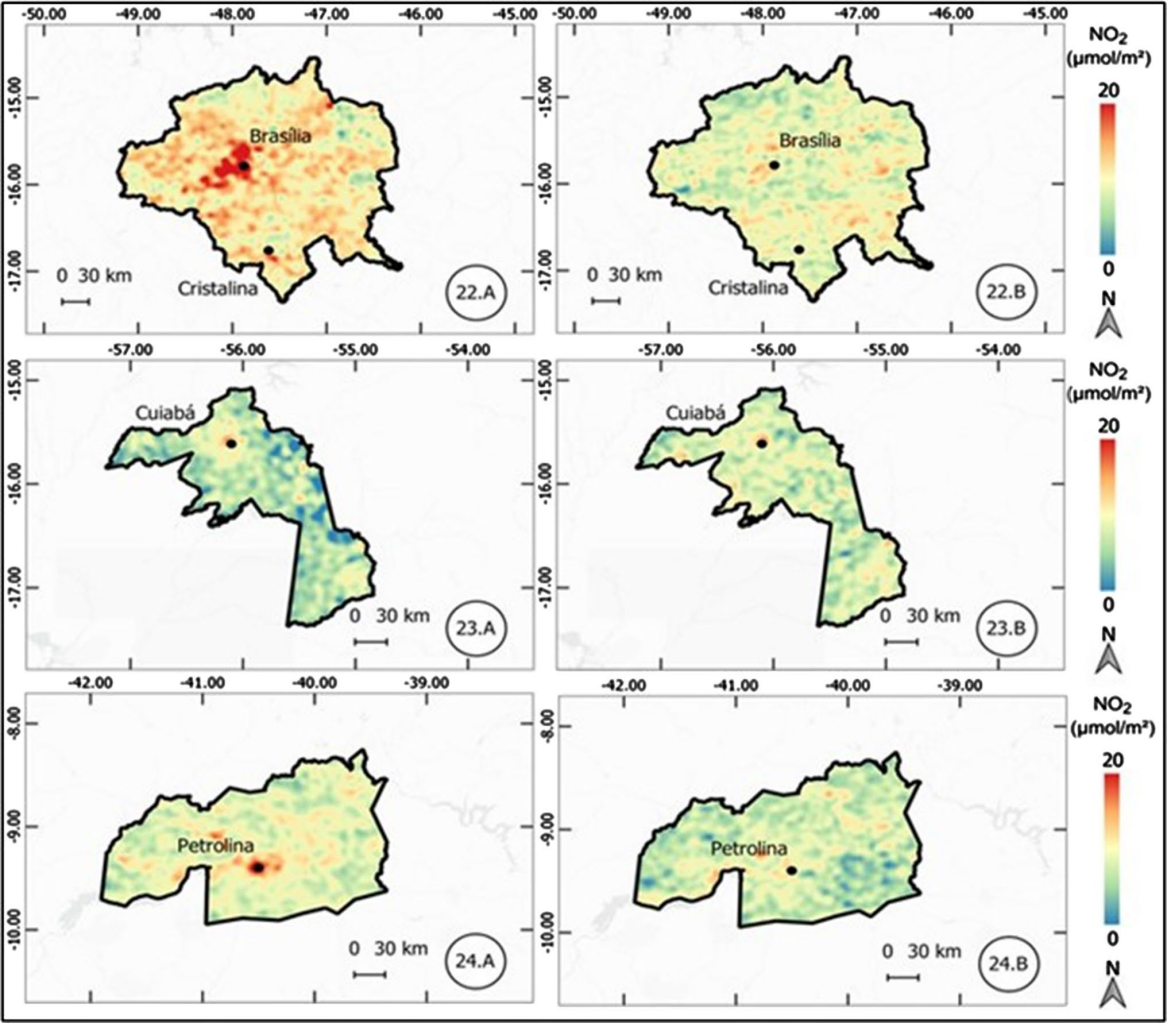
Thematic maps of the mean concentration of NO_2_ from March 12 to April 16, 2019 and 2020, in the metropolitan regions of Brasília (Federal District), Cuiabá, and Petrolina

The results of the atmospheric concentration of NO_2_ are presented in the form of thematic maps (Figs. 1, 2, 3, 4, 5, 6, and 7), which demonstrate the variation of NO_2_ in all metropolitan regions studied, and also in Table 1, which synthesizes the results obtained and presents the final result, in terms of percentage variation, of the two periods analyzed.

**Table 1:** Mean concentration of NO_2_ in metropolitan regions, from March 12 to April 16, 2019 and 2020

Metropolitan Region	Period in 2019		Period in 2020		Average reduction NO_2_ (%)
	Mean (µmol m^−2^)	Standard Deviation (µmol m^−2^)	Mean (µmol m^2^)	Standard Deviation (µmol m^−2^)	
Baixada Santista	41.2	27.7	21.1	10.9	48.7
São Paulo	57.2	39.8	35.6	20.4	37.9
Salvador	17.9	5.8	12.1	3.7	32.5
Florianópolis	13.2	3.9	9.2	2.2	30.3
Rio de Janeiro	32.2	20.1	23.6	10.1	26.6
Vale do Paraíba	16.9	6.6	12.8	5.3	24.1
Recife	16.9	4.5	12.8	1.8	24.1
Sorocaba	22.6	8.9	17.7	5.3	21.6
Campinas	30.2	8.5	24.2	6.4	20.0
Distrito Federal	12.0	2.6	9.8	2.0	18.8
Fortaleza	12.4	3.7	10.2	2.0	17.8
Curitiba	16.4	8.0	13.5	5.3	17.5
Goiânia	13.7	3.7	11.4	3.3	16.6
Belo Horizonte	16.6	5.8	14.7	4.5	11.3
São Luis	11.1	2.3	9.8	3.0	11.2
Petrolina	11.2	1.7	10.3	1.8	8.6
Natal	9.6	1.6	8.9	1.1	7.4
Maceió	11.4	1.4	10.8	1.5	5.3
Manaus	6.2	1.6	5.8	1.6	5.2
Teresina	10.0	1.4	9.7	1.6	3.7
Belém	8.2	2.4	8.0	3.3	2.6
Vitória	19.9	7.4	20.2	5.9	-1.7
Porto Alegre	16.0	4.6	16.6	4.4	-3.9
Cuiabá	9.1	2.1	10.6	1.7	-15.7

### Atmospheric concentration of NO_2_

#### Baixada Santista metropolitan region

Baixada Santista metropolitan region, located on the coast of the state of São Paulo, showed a 48.7% reduction in the concentration of NO_2_ in the comparison between the period of 2019 (left) and 2020 (right). This was the highest percentage reduction observed in the metropolitan regions that were object of this study.

As shown in Fig. 1, in 2020 the high concentrations of NO_2_ were limited to the urban and industrial nucleus of the cities of Cubatão and Santos, unlike the period of 2019, where the high concentrations were observed more than 40 km from these cities.

#### Belém, Belo Horizonte, Campinas, and Curitiba metropolitan regions

Belém metropolitan region, located in northern Brazil, presented a little significant reduction of 2.6% in the concentration of NO_2_ in the two periods studied. The map in Fig. 2 shows a reduction in concentration on the urban center of Belém, compensated by the increase in other areas.

In Belo Horizonte metropolitan region, located in southeastern Brazil, the reduction observed was 11.3%, even though it was delimited in a polygon of larger scale. Then, we observe a significant reduction of 20% in the region of Campinas, which had high concentrations, in 2019, between Campinas and Americana, not observed in the period of 2020.

Finally, the reduction is also substantial in the metropolitan region of Curitiba, located in the Southern region of Brazil, where there was a decrease of 17.5%.

#### Florianópolis, Fortaleza, Goiânia, and Maceió metropolitan regions

In the metropolitan region of Florianópolis, in southern Brazil, there was an expressive reduction of 30.3% in the concentration of NO_2_. As shown in Table 1, it is possible to notice, in addition to the reduction of the mean concentration, a reduction of the standard deviation of the obtained values, even though the values of concentrations are considered low. The metropolitan regions of Fortaleza and Maceió, both located in northeastern Brazil, had a decrease of 17.8% and 5.3%, respectively. These results can be verified in Fig. 3.

#### Manaus, Natal, Porto Alegre, and Recife metropolitan regions

The metropolitan region of Manaus, located in the state of Amazonas, northern Brazil, showed a small reduction in atmospheric NO_2_ concentration; however, this reduction of only 5.2% is overshadowed by the scale of the metropolitan region, which covers a polygon with thousands of square kilometers, as illustrated in Fig. 4. The metropolitan regions of Natal and Recife, both located in northeastern Brazil, had a reduction of 7.4 and 24.1%, respectively. On the other hand, the metropolitan region of Porto Alegre, located in southern Brazil, had an increase of 3.9% in the concentration of NO_2_, different from results observed so far.

#### Rio de Janeiro, Salvador, São Luís, and São Paulo metropolitan regions

In the metropolitan region of Rio de Janeiro, located in the state of the same name and southeastern region of Brazil, there was a significant reduction in NO_2_ concentration, 26.6%. In this case, the substantial reduction was observed in both the mean concentration of NO_2_ and the standard deviation when comparing the results of 2019 and 2020.

In the metropolitan regions of Salvador and São Luís, both located in the northeast region, there was a reduction of 32.5 and 11.2%, respectively. Figure 5 demonstrates the intense reduction observed in the region known as Recôncavo Bahiano.

Finally, the metropolitan region of São Paulo, the largest in the country, also located in the southeast region, had the second largest percentage (37.9%) reduction among the metropolitan regions studied.

#### Sorocaba, Teresina, São José dos Campos (Vale do Paraíba), and Vitória metropolitan regions

In the metropolitan region of Sorocaba, located in the state of São Paulo, the reduction of NO_2_ in the compared periods was 21.6%. Figure 6 shows a reduction in the entire length between the cities of Sorocaba and Itu. Also, in the state of São Paulo, the metropolitan region of Vale do Paraíba recorded a reduction of 24.1%, more significant in the urban nucleus of São José dos Campos. Still in the southeast, there was no significant change in the metropolitan region of Vitória, Espírito Santo state. In this region, there was an increase of 1.7% in the mean atmospheric concentration of NO_2_, although there was a reduction, according to Fig. 6, on the urban centers of Vitória and, mainly, Vila Velha.

The metropolitan region of Teresina, located in the northeast, also showed low variation in the atmospheric concentration of NO_2_.

#### Brasília (Federal District), Cuiabá, and Petrolina metropolitan regions

A percentage reduction of 18.8% was observed in the Distrito Federal (Federal District of Brazil), in the Brazilian Midwest region. Although the reduction is significant, the concentration values are considered low in both periods, as shown in Fig. 7. The region of Petrolina, in the northeast, showed a reduction of 8.6%.

**Fig. 8:**
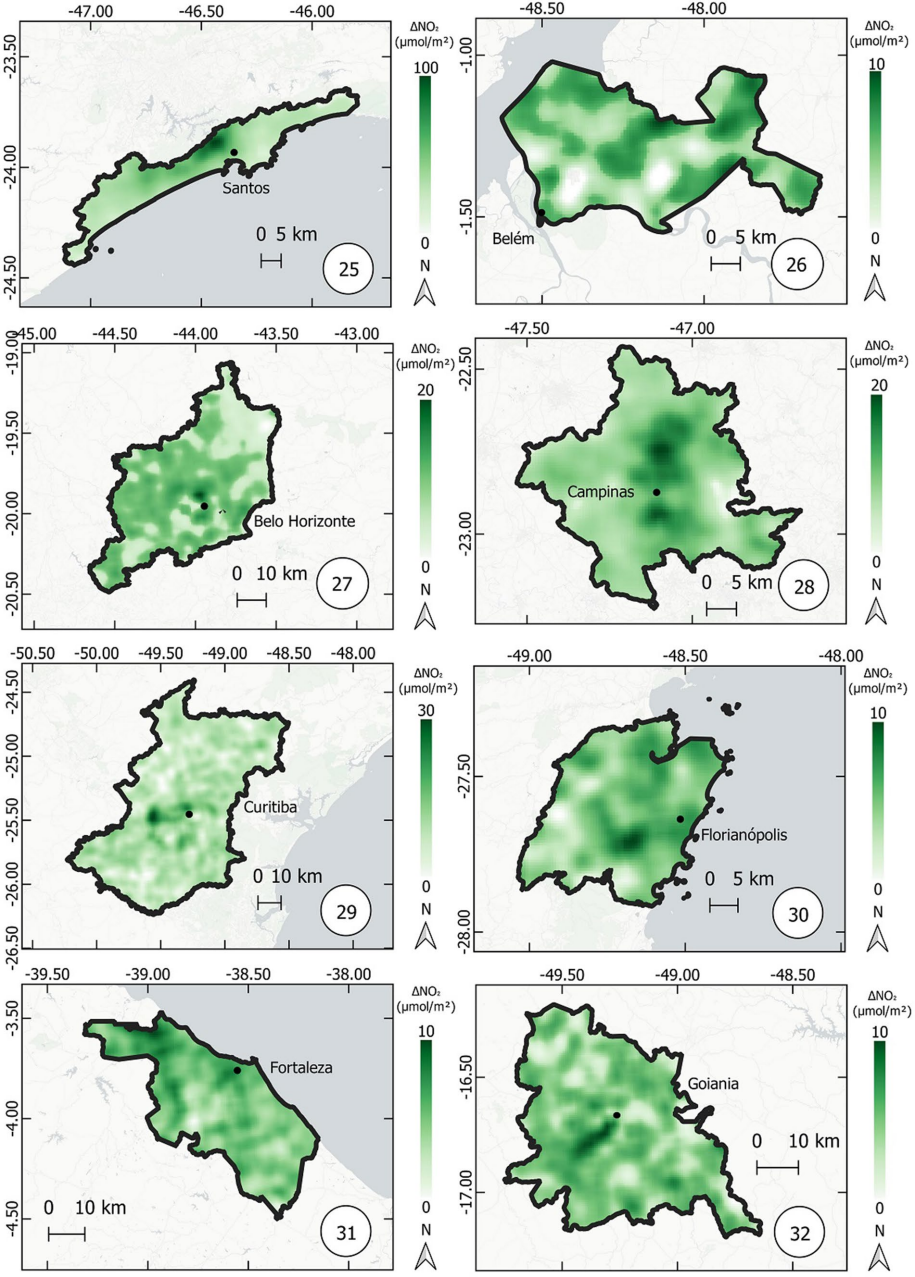
Thematic maps of reduction in NO_2_ concentrations from March 12 to April 16, 2019 and 2020, in the metropolitan regions of Baixada Santista, Belém, Belo Horizonte, Campinas, Curitiba, Florianópolis, Fortaleza, Goiânia,

**Fig. 9:**
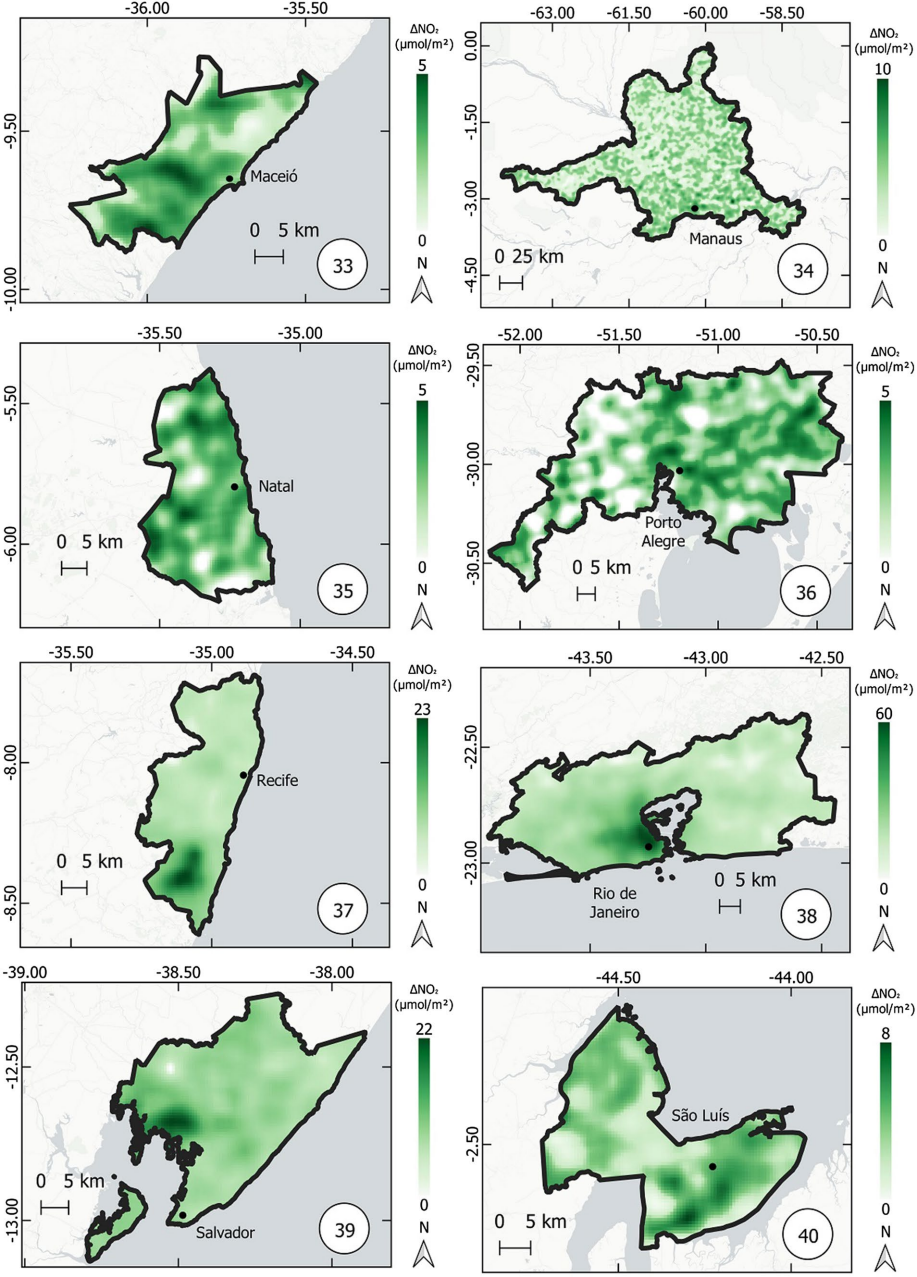
Thematic maps of reduction in NO_2_ concentrations from March 12 to April 16, 2019 and 2020, in the metropolitan regions of Maceió, Manaus, Natal, Porto Alegre, Recife, Rio de Janeiro, Salvador e São Luís,

**Fig. 10:**
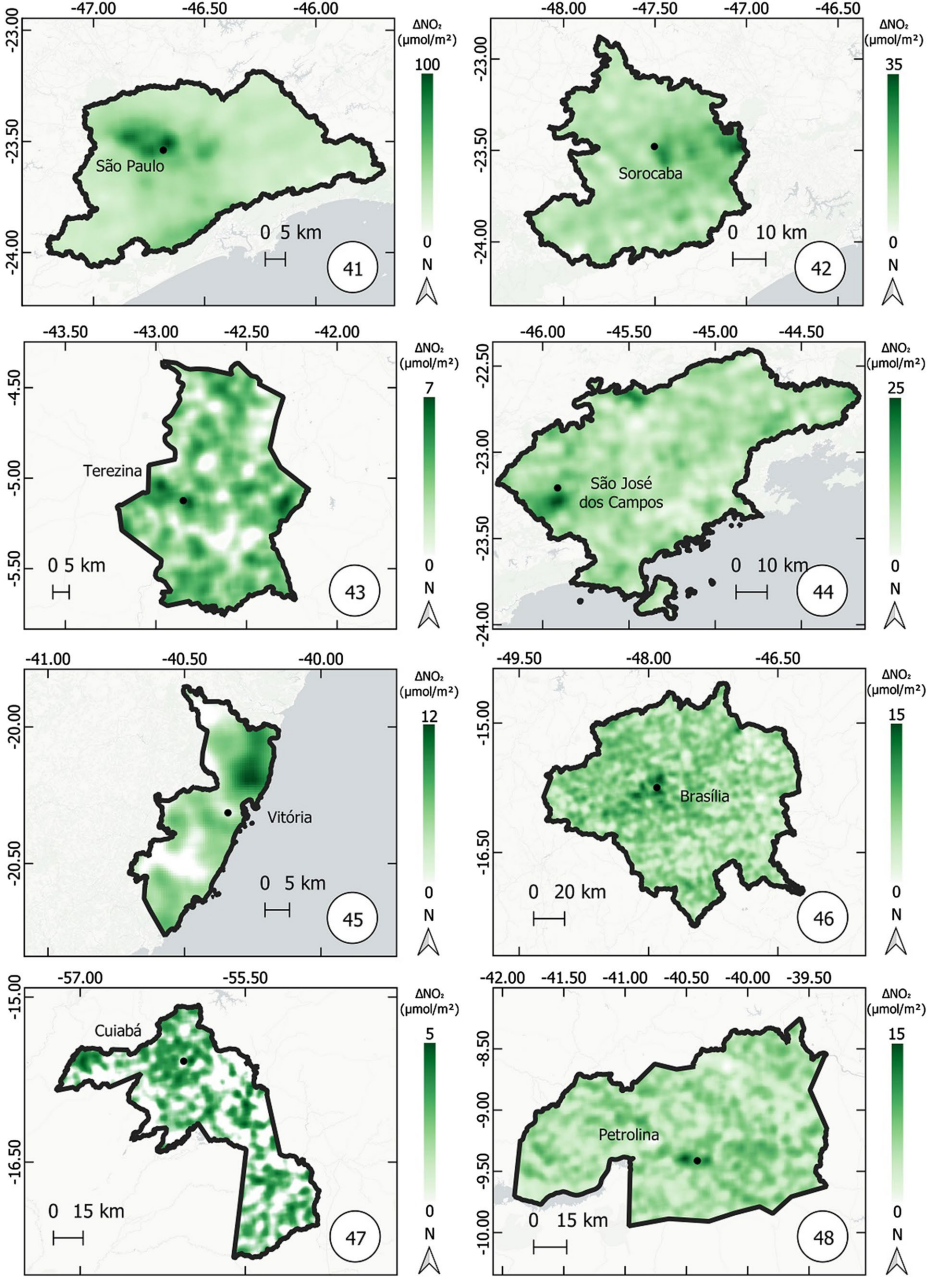
Thematic maps of reduction in NO_2_ concentrations from March 12 to April 16, 2019 and 2020, in the metropolitan regions of São Paulo, Sorocaba, Teresina, São José dos Campos, Vitória, Brasília, Cuiabá, Petrolina,

Finally, the metropolitan region of Cuiabá, also in the Midwest, registered an increase of 15.7% in 2020 compared to 2019, a movement in the opposite direction to that observed in most metropolitan regions. It is observed, however, that like Manaus MR, the MR scale can distort the results, since the urban center of Cuiabá occupies only a small fraction of the polygon.

Figures 8, 9, and 10 show the variation in NO_2_ concentration (only reduction) in all metropolitan regions. The higher the reduction, the higher the intensity of green color.

Table 1 summarizes the results obtained by the model for the evaluation of NO_2_ concentration in the Brazilian metropolitan regions evaluated.

It is possible to observe that in 21 (87.5%) of the 24 metropolitan regions there was a reduction in the mean concentration of atmospheric NO_2_. Even in regions with no reduction, one can verify the effect of the scale of the metropolitan region on the result, as occurred in Cuiabá. Similarly, in Vitória MR, there was a significant reduction in the urban centers of Vitória and Vila Velha; however, the result was affected by the adjacent regions and not necessarily in inhabited areas, although within the metropolitan region.

### Variation of NO_2_correlations

The reduction of NO_2_ in the comparison between the two periods was observed in most of the metropolitan regions. However, being a continental country, the Brazilian metropolitan regions have different socioeconomic characteristics, such as territorial extension, population, and number of vehicles circulating in the regions.

Table 2 indicates the results of Spearman's correlation between the variation in NO_2_ concentration and the population density (number of inhabitants per square kilometer), the density of vehicles (number of vehicles per square kilometer), and the variation in O_3_ concentration (in %, in the comparison between 2020 and 2019) in metropolitan regions.

**Table 2:** Spearman’s correlations for NO_2_ variation_,_ O_3_ variation, population density, and vehicle density

Spearman’s Rho		NO_2_ var (%)	O_3_ var (%)	Population density	Vehicle density
NO_2_ var (%)	Correlation Coefficient	1.000	− 0.388	0.424^*^	0.485^*^
	Sig. (2-tailed)		0.061	0.039	0.016
	N	24	24	24	24
O_3_ var (%)	Correlation Coefficient	− 0.388	1.000	− 0.034	− 0.271
	Sig. (2-tailed)	0.061		0.875	0.200
	N	24	24	24	24
Population density	Correlation Coefficient	0.424^*^	-0.034	1.000	0.922^**^
	Sig. (2-tailed)	0.039	0.875		0.000
	N	24	24	24	24
Vehicle density	Correlation Coefficient	0.485^*^	− 0.271	0.922^**^	1.000
	Sig. (2-tailed)	0.016	0.200	0.000	
	N	24	24	24	24

^*^Correlation is significant at the 0.05 level (2-tailed); **Correlation is significant at the 0.01 level (2-tailed)

At a significance level of 0.05, a moderate positive correlation (Spearman’s Rho = 0.485) was found between the NO_2_ variation and the vehicle density. This means that in the evaluated metropolitan regions, as the density of vehicles increases, a reduction in the concentration of atmospheric NO_2_ is expected. The existence of a positive moderate correlation was also verified for population density (Spearman’s Rho = 0.424). These are indications that point to the relevance of the contribution of combustion engines to air quality in metropolitan regions.

Regarding O_3_, as the p-value is above the 0.05 significance level, the association between variations in the concentration of NO_2_ and O_3_ was inconclusive, although the correlation coefficient shows a moderate negative correlation.

## Discussion

Our analysis disclosed that satellite images showed a reduction in average NO_2_ concentrations in 21 Brazilian metropolitan regions between March 12 and April 16, 2020, compared to the same period in 2019. Table 1 indicates a reduction in the average concentrations of NO_2_ of 48.7% in the metropolitan region of Baixada Santista, a place with the greatest reduction, followed by the region of São Paulo (37.8%) and Salvador (32.5%). In São Paulo, the reduction observed in this study for the concentration of NO_2_ is in line with the results obtained by automatic air quality monitoring stations, which revealed a 25% reduction in concentration in March 2020 when compared to March from 2015 to 2019 (Connerton et al. 2020). Likewise, a reduction of 26.6% found in this study for the metropolitan region of Rio de Janeiro is similar to the obtained in the evaluation by two automatic NO_2_ monitoring stations, which between 23 March and 12 April during the years 2019 and 2020 experienced a reduction of 32.9% and 24.1% in the concentration of the pollutant (Dantas et al. 2020).

In the state of Rio Grande do Sul, the State Foundation for Environmental Protection (FEPAM) issued a report concluding that there were no significant changes in the concentration of NO_2_ in the month of April 2020 when compared to the years 2017 to 2019, obtained by the automatic monitoring stations of Esteio, Guaíba, and Gravataí, all located in the Metropolitan Region of Porto Alegre (DQA 2020). In this work was verified a small increase of 3.6% in the concentration of NO_2_ in the MR, confirming the insignificant variation observed by the environmental agency.

In the state of Minas Gerais, the State Environment Foundation published the report entitled “Impacts on air quality after stoppage of activities due to the COVID-19 pandemic.” The report presents significant reductions in the concentration of PM_2.5_ and PM_10_ in the periods of 20 March and 20 April in 2019 and 2020. There are two automatic monitoring stations in the metropolitan region of Belo Horizonte. The first showed a reduction of 31% in the concentration of PM_10_ and 45% in the concentration of PM_2.5_, while the second station pointed out a reduction of 10% for PM_10_ and 26% for PM_2.5_ (Belo Horizonte 2020). There is no diagnosis for NO_2_, but the report points to a 74% reduction in traffic circulation in Belo Horizonte (2020), indicating a strong tendency to reduce NO_2_, as was verified in this study.

To date, no other published scholarly studies, to our knowledge, found that portrayed the variation in the concentration of air pollutants in other Brazilian metropolitan regions, using automatic air quality monitoring stations as a source of data. Nonetheless, as demonstrated, the existing ones confirm the results obtained in this study.

This reduction may be associated with the Brazilian government decrees recommending social isolation in addition to quarantine as a non-pharmacological measure for coping with coronavirus, which occurred, in general, from March 14, 2020 (Brasil 2020). In some states, the decrees were issued before that date, as in Alagoas (13 March), Distrito Federal (11 March), São Paulo (13 March), which stands out among the findings, because 5 out of the 21 regions that presented reduction in the emission of NO_2_ are in its territory. These results explain the perception of improved air quality reported by Brazilian people, where 934 citizens, 60% of the state of Minas Gerais, 21.6% from the state of São Paulo, 3.7% from Rio de Janeiro, 2.4% from Bahia, and 2.3% from Federal District, which makes up more than 90% of the sample, reported a better quality of breathed air (Barbieri et al 2020). These five states have nine metropolitan regions portrayed by this study, and in all of them there was a reduction in the concentration of atmospheric NO_2_.

This evidence is not only pointed out by Brazil. China also showed a reduction in the average concentration of NO_2_, recording a decrease in some of its cities, especially in February 2020, when the government has imposed interdiction measures to contain the Covid-19 epidemic (Chen et al. 2020, 2021; Dutheil et al. 2020; Venter et al. 2020; Wang et al. 2020). Similar situation was also detected in Europe (Ogen 2020; Liu et al. 2021; Burns et al. 2021; Jephcote et al. 2021; Wyche et al. 2021), Asia (Mahato et al. 2020; Thomas et al. 2020; Tyagi et al. 2021), and the USA (Connerton et al. 2020; Shi et al. 2021).

This drop coincides with the recommendation of quarantine and isolation, which limits road transport, considered the largest unnatural source of NO_2_, and decreases the production of factories and industries and consequent daily burning of fossil fuels that causes a high concentration of atmospheric NO_2_ (Derísio 2017; He et al. 2020; Chen et al. 2021). In addition to the smaller number of vehicles in circulation, freer traffic conditions and the absence of traffic jams also contribute to a lower emission of pollutants.

The study at hand also investigated the effects of population and vehicle density on the percentage variation of NO_2_. Considering that social isolation measures were initiated at approximately the same time (second half of March) in all metropolitan regions, that a reduction in concentration was observed and the population and vehicle fleet remained relatively stable during the period, it was possible to observe the indirect effects of population and vehicle density in the reduction of NO_2_ concentration. At a significance level of 0.05, the Spearman correlation coefficient found was 0.424 (population density) and 0.485 (vehicle density). The results indicate that there seems to be a dependency, albeit indirect, between the variables. This means that in those metropolitan regions where there are more vehicles per square kilometer there is a tendency for a more accentuated variation in the concentration of NO_2_. Higher vehicle densities may be related to a greater number of NO_2_ emitting hot spots, such as high-flow highways or roads and traffic jams.

This measure of concentration of pollutants in the air can be an important public health factor, because as pollution levels decrease, there is a 6% reduction in mortality cases (39). Considering that the interaction of the human being with the environment that is lived produces an important part of emissions that lead to air pollution (Derísio 2017), the evidence of this study shows that attitudes of social isolation directly interfere in the reduction of NO_2_ emission that, consequently, will interfere in other important health indicators.

This is because data from the WHO show that more than four million people died worldwide from respiratory diseases per year (Cohen et al. 2017), caused by air pollution, not to mention preventable non-transmissible diseases, which may also be associated with this factor (Neira et al. 2018; Chen and Bloom 2019).

The pandemic issue sparks an important debate about the need to rethink city designs, looking at them from the perspective of health, habitability, and sustainability (Guaralda et al. 2020). In Italy, for instance, the highest incidence of cases and deaths related to Covid-19 was found in cities known to be more polluted. In cores characterized by the use of renewable energies, with generation of predominantly wind energy, there was a slowdown in the incidence of Covid-19 (Coccia 2020). The high concentration of air pollutants in urban centers exposes the weaknesses of urban centers and that puts the health of the population at risk. In Dhaka and Brisbane, for instance, the most susceptible areas to the direct impacts of climate change over the time are the affected by the peri-urbanization (Mortoja and Yigitcanlar 2020a, b).

The significant variations in NO_2_ concentration in the metropolitan regions of Brazil also expose the need to reassess Brazil’s urban centers, since they demonstrate the emission of gases harmful to health and the environment and open wide that, although there have been regulatory instruments and development policies for the same time, there is a need for improvements in its effective implementation (Sotto et al. 2019). Encouraging the use of public transport and changing the energy matrix, with the gradual replacement of combustion vehicles, for example, are initiatives that can and should be encouraged, with instant results in air quality, as observed in the regions metropolitan areas that, forcibly, drastically reduced emissions from motor vehicles (Arbolino et al. 2018; Kamruzzaman et al. 2015).

Thus, in view of the direct relationship between the concentration of atmospheric NO_2_ and pollution, this study can enhance the sustainable resumption of production linked to the emission of precursor gases of NO_2_, despite the protection of pollution control measures, which directly reflect on the living and health conditions of the population in general, since contingency measures demonstrated a substantial decrease of NO_2_ concentration levels.

It is, hence, critical to develop sound strategies for not to reverse the air quality improvements achieved during the pandemic years. In other words, stringent government policies are needed to keep the emissions low and even further lower them (Rivers and Jaccard 2005; Pane 2020; Dang and Trinh 2021). To this end, we advocate the following actions for government authorities to adopt:

- Putting a cost on pollution by pricing the carbon externality for both producers and users of the polluting goods or services (Sterner 2012; Hagmann et al. 2019);
- Providing incentives for the development and use of sustainable energy and mobility solutions (Butler et al. 2020, 2021);
- Revisiting climate and energy-related legislations and adopting the New Green Deal for speeding up a green transition (Hafner and Raimondi 2020; Barry and Hoyne 2021);
- Investing on the state-of-the-art air quality monitoring and analysis systems (Liu et al. 2018; Gonzalez-Martin et al. 2021)
- Planning for sustainable urban form and transport integration to reduce emissions (Yigitcanlar and Kamruzzaman 2014; Dur and Yigitcanlar 2015);
- Offering government-financed stimuli for not rolling back environmental regulations and halting spending on a green transition (Elliott et al. 2020; D’Orazio 2021);
- Initiating the adoption of degrowth model all across the globe for reduced emissions (Perkins 2019; Kallis 2021);
- Depoliticizing the climate change and making climate action inclusive of all sides of politics and all members of the public through education and awareness raising campaigns (Díaz‐Pont 2021; Mortoja and Yigitcanlar 2022).

## Conclusion

The measures adopted to contain the Covid-19 virus (SARS-CoV-2) pandemic showed a reduction in the emission of NO_2_ in most metropolitan regions of Brazil, especially after the state of pandemic, determined by WHO, which in turn decreased fuels burning due to the reduction of circulation of vehicles and industrial activities, to the detriment of social isolation and quarantine measures encouraged to reduce the transmission of SARS-CoV-2.

This study raises the government’s need to encourage public policies that enable a rational organization about economic activities and the preservation of the environment in the post-pandemic period, since such actions impact on the reduction of mortality rates from other diseases respiratory, since the increase in NO_2_ causes toxicity when there is long-term exposure, in the maintenance of air quality and the planet’s biodiversity, because the high level of this compound in the air leads to damage to nature due to its corrosive characteristic, causing acid rain which, when falling to the surface, alters the chemical composition of the soil and water, reaching food chains, destroying forests and crops.

Therefore, this research leads to reflection on new strategies that are alternatives to the reduction of NO_2_ emission, such as accessible public transport, use of alternative energy sources, such as electricity, solar, biofuel, to maintain the health of the population.

In this regard, we conclude the paper by advocating Quéré et al. (2021) following assertion, “five years after the adoption of the Paris Climate Agreement, growth in global CO_2_ emissions has begun to falter. The pervasive disruptions from the Covid-19 pandemic have radically altered the trajectory of global CO_2_ emissions. Contradictory effects of the post-Covid-19 investments in fossil fuel-based infrastructure and the recent strengthening of climate targets must be addressed with new policy choices to sustain a decline in global emissions in the post-Covid-19 era.” As emphasized by Sarimin and Yigitcanlar (2012) one of the promising policy choices to achieve climate and sustainable development targets is the knowledge-based development of our cities and societies.

Besides the NO_2_ findings, our study has also underlined the importance of continuous, i.e., time series, analysis on the air quality. Moreover, in interpretation of the results, we note the following limitations of the study. The limitations of this case report are tied to the period researched (the period of 36 days, 12 March to 16 April both in 2019 and 2020), since we considered the WHO declaration of pandemic as the start date for the execution of the research in all regions. Nonetheless, there is a delay between that declaration and the restriction measures adopted by governments and regional agencies. Furthermore, investigation of a single air pollutant is a limitation, despite we also liked at the O_3_. Another limitation of this study is not to focus on the policy issues and prescribe public policy and actions to address the air pollution and climate change issues in Brazil. Nevertheless, our prospective research will focus on these limitations by considering NO_2_ and also other pollutants and investigating larger timeframes and conduct time series analyses and prescribe relevant policy actions.

## Data Availability

All data are available in its sources.

## Abbreviations

API: Application programming interface
CH_4_: Methane
CO: Carbon monoxide
CO_2_: Carbon dioxide
DENATRAN: Brazilian Transportation Department
ESA: European Space Agency
FEPAM: State Foundation for Environmental Protection
HCHO: Formaldehyde
IBGE: Brazilian Institute of Geography and Statistics
IPEA: Institute of Applied Economic Research
MR: Metropolitan region
NIR: Near infrared
NO_2_: Nitrogen dioxide
NRTI: Near real-time high-resolution imagery
O_3_: Ozone
PM_10_: Particulate matter—diameter of 10 microns or less
PM_2,5_: Particulate matter—diameter of 2.5 microns or less
SO_2_: Sulfur dioxide
SPSS: Statistical Package for the Social Sciences
SWIR: Short-wave infrared
TROPOMI: Tropospheric Monitoring Instrument
UV: Ultraviolet
VIS: Visible
WHO: World Health Organization

## Code availability

(software application or custom code). All softwares are open source. The created code is attached.
